# Amino acid and lipid metabolism in post-gestational diabetes and progression to type 2 diabetes: A metabolic profiling study

**DOI:** 10.1371/journal.pmed.1003112

**Published:** 2020-05-20

**Authors:** Mi Lai, Ying Liu, Gabriele V. Ronnett, Anne Wu, Brian J. Cox, Feihan F. Dai, Hannes L. Röst, Erica P. Gunderson, Michael B. Wheeler

**Affiliations:** 1 Department of Physiology, Faculty of Medicine, University of Toronto, Toronto, Ontario, Canada; 2 Metabolism Research Group, Division of Advanced Diagnostics, Toronto General Research Institute, Toronto, Ontario, Canada; 3 Janssen Research & Development, World Without Disease Accelerator, Spring House, Pennsylvania, United States of America; 4 Donnelly Centre for Cellular and Biomolecular Research, University of Toronto, Toronto, Ontario, Canada; 5 Division of Research, Kaiser Permanente Northern California, Oakland, California, United States of America; Chinese University of Hong Kong, CHINA

## Abstract

**Background:**

Women with a history of gestational diabetes mellitus (GDM) have a 7-fold higher risk of developing type 2 diabetes (T2D) during midlife and an elevated risk of developing hypertension and cardiovascular disease. Glucose tolerance reclassification after delivery is recommended, but fewer than 40% of women with GDM are tested. Thus, improved risk stratification methods are needed, as is a deeper understanding of the pathology underlying the transition from GDM to T2D. We hypothesize that metabolites during the early postpartum period accurately distinguish risk of progression from GDM to T2D and that metabolite changes signify underlying pathophysiology for future disease development.

**Methods and findings:**

The study utilized fasting plasma samples collected from a well-characterized prospective research study of 1,035 women diagnosed with GDM. The cohort included racially/ethnically diverse pregnant women (aged 20–45 years—33% primiparous, 37% biparous, 30% multiparous) who delivered at Kaiser Permanente Northern California hospitals from 2008 to 2011. Participants attended in-person research visits including 2-hour 75-g oral glucose tolerance tests (OGTTs) at study baseline (6–9 weeks postpartum) and annually thereafter for 2 years, and we retrieved diabetes diagnoses from electronic medical records for 8 years. In a nested case–control study design, we collected fasting plasma samples among women without diabetes at baseline (*n =* 1,010) to measure metabolites among those who later progressed to incident T2D or did not develop T2D (non-T2D). We studied 173 incident T2D cases and 485 controls (pair-matched on BMI, age, and race/ethnicity) to discover metabolites associated with new onset of T2D. Up to 2 years post-baseline, we analyzed samples from 98 T2D cases with 239 controls to reveal T2D-associated metabolic changes. The longitudinal analysis tracked metabolic changes within individuals from baseline to 2 years of follow-up as the trajectory of T2D progression. By building prediction models, we discovered a distinct metabolic signature in the early postpartum period that predicted future T2D with a median discriminating power area under the receiver operating characteristic curve of 0.883 (95% CI 0.820–0.945, *p* < 0.001). At baseline, the most striking finding was an overall increase in amino acids (AAs) as well as diacyl-glycerophospholipids and a decrease in sphingolipids and acyl-alkyl-glycerophospholipids among women with incident T2D. Pathway analysis revealed up-regulated AA metabolism, arginine/proline metabolism, and branched-chain AA (BCAA) metabolism at baseline. At follow-up after the onset of T2D, up-regulation of AAs and down-regulation of sphingolipids and acyl-alkyl-glycerophospholipids were sustained or strengthened. Notably, longitudinal analyses revealed only 10 metabolites associated with progression to T2D, implicating AA and phospholipid metabolism. A study limitation is that all of the analyses were performed with the same cohort. It would be ideal to validate our findings in an independent longitudinal cohort of women with GDM who had glucose tolerance tested during the early postpartum period.

**Conclusions:**

In this study, we discovered a metabolic signature predicting the transition from GDM to T2D in the early postpartum period that was superior to clinical parameters (fasting plasma glucose, 2-hour plasma glucose). The findings suggest that metabolic dysregulation, particularly AA dysmetabolism, is present years prior to diabetes onset, and is revealed during the early postpartum period, preceding progression to T2D, among women with GDM.

**Trial registration:**

ClinicalTrials.gov Identifier: NCT01967030.

## Abstract 2

Mi Lai and co-workers describe a metabolic signature associated with progression from gestational to type 2 diabetes.

## Author summary

**Why was this study done?:**

- Women with a history of gestational diabetes mellitus (GDM) have a 7-fold higher risk of developing type 2 diabetes (T2D) later in life, and an estimated 35%–50% of GDM cases will progress to T2D within 10 years postpartum. Biological pathways and metabolites influencing progression from GDM to T2D have not been elucidated in humans.
- The main goal of the present work is to gain critical insight into the pathology of the transition from GDM to T2D, particularly metabolic changes involved in this process. A second goal is to devise more accurate means of identifying who will transition to T2D among women with GDM.

**What did the researchers do and find?:**

- We carried out a nested case–control study using a GDM prospective cohort of 1,010 women without T2D 6–9 weeks postpartum (study baseline). We performed metabolic profiling on fasting blood samples from women at 6–9 weeks postpartum (baseline) and up to 2 years post-baseline (follow-up).
- We found that significant dysmetabolism in lipids and amino acids in the early postpartum period was associated with future diabetes in women. Longitudinal analysis tracking changes in metabolic profiles over the course of diabetes progression revealed 10 specific metabolites that are associated with progression to T2D.
- We identified a small group of metabolites that could predict future T2D with great discriminative power, surpassing current clinical methods.

**What do these findings mean?:**

- A subclinical diabetes-like condition appears to already be present in the early postpartum period in women with previous GDM who later progress to T2D, and dysregulated amino acid metabolism is tightly associated with disease progression.
- Metabolites other than glucose may provide a simple and accurate alternative approach to assess risk of future T2D in women with a history of GDM.

## Introduction

Gestational diabetes mellitus (GDM) is defined as glucose intolerance that is first recognized during pregnancy; it occurs in approximately 7%–8% of pregnant women [1], although different estimates range up to 20% for milder forms based on the diagnostic criteria [2]. In the vast majority of cases, women return to normoglycemia post-delivery, but up to 35% may have impaired glucose tolerance within 2 months post-delivery [3]. Women with a history of GDM have a 7-fold higher risk of developing type 2 diabetes (T2D) than women without previous GDM [1,4,5]. In fact, an estimated 35%–50% of GDM cases will progress to T2D within 10 years postpartum [4,6]. These women not only develop T2D at a relatively younger age (e.g., <40 years) compared to women in general, but are also more likely to develop non-alcoholic fatty liver and cardiovascular and renal diseases that may lead to early mortality [7–15]. For these reasons, it is important to develop an accurate means to predict the future transition from GDM to T2D after pregnancy, and to gain a better understanding of the distinct pathophysiology of the metabolic disturbances preceding the progression to T2D and its underlying causes for the GDM population.

Currently, the recommended test to reclassify glucose tolerance and assess future T2D risk after GDM pregnancy is the 2-hour 75-g oral glucose tolerance test (OGTT) at 6 to 12 weeks postpartum followed by subsequent testing for diabetes every 1–3 years via fasting plasma glucose (FPG) and 2-hour OGTT [16]. However, the accuracy of 2-hour 75-g OGTT for prediction of future T2D ranges from 65% to 77% [17–19]. Moreover, adherence to the American Diabetes Association (ADA) recommendations to undergo the 2-hour OGTT after pregnancy is generally very low, at approximately 20% [19,20], and in most settings, fewer than 30% of women with GDM complete the recommended testing for reclassification of glycemia during the postpartum period [19,21–23]. For these reasons, a more convenient and accurate predictive test is needed to assess glucose tolerance and for early prediction of future progression to overt diabetes following GDM delivery.

Discovery-based metabolomics have recently revealed specific metabolites in addition to glucose that can facilitate the early prediction of T2D in the general population. For example, branched-chain amino acids (BCAAs) have been identified as a putative biomarker for future T2D incidence [24–26]. Our group has previously identified metabolic markers of future T2D among women with recent GDM [19]. Using clinical variables combined with metabolic biomarkers, including lipid species, our simple metabolic signature [PCaeC40:5, hexoses, BCAAs, and SM(OH)C14:1] predicted T2D incidence with an approximate 80% area under the receiver operating characteristic curve (AUC) in a nested case–control study of 122 matched pairs (matched on race/ethnicity, age, and BMI) identified from the Study of Women, Infant Feeding and Type 2 Diabetes after GDM Pregnancy (SWIFT). SWIFT is a prospective cohort of 1,035 women with GDM tested at 6–9 weeks post-delivery (study baseline) and annually thereafter for up to 2 years post-baseline for new onset T2D [19]. Another smaller study using targeted measurements of >300 lipid species (lipidomics) in blood samples collected 12 weeks post-delivery from 104 women with GDM, 21 of whom developed T2D, showed 84% accuracy in T2D prediction based on 3 lipids [i.e., PE(P-36:2), PS38:4, CE20:4] in combination with 6 other risk factors (i.e., age, BMI, pregnancy FPG, postpartum FPG, total triglycerides, and total cholesterol) [27]. In a recent purely lipidomic analysis, we identified a 7-metabolite predictive signature with a discriminating power of 92% for prediction of incident T2D after GDM among a subset of Asian and Hispanic matched pairs from the prospective SWIFT cohort [28]. These promising findings warrant further investigation, and suggest that novel metabolite markers combined with known clinical parameters may provide more accurate early risk prediction for future T2D in the GDM population compared to current testing regimens.

Metabolomics is also a valuable tool to better understand the pathophysiology of the transition from GDM to T2D. Recently, our team found that short-chain acylcarnitines were associated with onset of T2D and had a negative impact on pancreatic beta cell function [29]. Other groups have demonstrated that BCAAs are increased preceding development of T2D among adults, potentially impairing insulin signaling and pancreatic beta cell function [25,30]. Several lipid metabolites have also been associated with future T2D risk. We recently showed that a decrease in specific sphingolipid species was linked with the transition from GDM to subsequent onset of T2D, and that reducing sphingolipid biosynthesis in beta cells impaired insulin secretion, suggesting a causal link between sphingolipids and insulin secretion [19]. Overall, these and other studies suggest the promise of using metabolomics as an approach to gain molecular insights into the transition (progression) from GDM to T2D and to identify metabolites other than carbohydrates that may contribute to longer-term diabetes risk.

In the present study, targeted metabolomics was used, analyzing fasting plasma samples from the SWIFT cohort obtained both at study baseline (6–9 weeks postpartum) and during 2 years of follow-up. We hypothesized that quantitative targeted metabolomics performed on blood samples from women who transition from GDM to T2D will reveal distinct metabolic changes associated with future T2D and that specific metabolites will have predictive value.

## Methods

We used the prospective SWIFT cohort in this study. The SWIFT cohort design, recruitment, selection criteria, and methodologies were conducted following a prospective study design and protocol (S1 Text). The bioinformatics and statistical analyses as described below were specifically developed for this analysis and did not follow an established protocol or analysis plan.

### SWIFT cohort

The SWIFT cohort is a prospective cohort that enrolled 1,035 racially and ethnically diverse (white, 23%; Asian, 36%; Hispanic, 31%; black, 8%; other, 2%) women (aged 20–45 years) who were diagnosed with GDM based on the 3-hour 100-g OGTT via Carpenter and Coustan criteria [31] and delivered a singleton, live-born infant after 35 weeks of gestation at a Kaiser Permanente Northern California (KPNC) hospital during the period 2008–2011 [32]. In the SWIFT cohort, 32.8% of participants were primiparous, 36.8% were biparous, and 30.4% were multiparous. Details of the study recruitment, selection criteria, and methodologies, and other detailed information have been described previously [32]. Briefly, participants were recruited from 13 KPNC medical center/office facilities in a 5,000-square-mile KPNC region from 10 September 2008 to 3 December 2011. Weekly, women recently diagnosed with GDM were added to a tracking system for the study. The selection criteria included women who had no history of diabetes or other serious health conditions, received standardized prenatal care, were not using medications affecting glucose tolerance, and were not planning another pregnancy or moving out of the area within the next 2 years. After eligibility pre-screening by authorized staff, around 2–4 weeks postpartum, potential participants were contacted by phone to determine their interest in participating in the study. Those who consented were invited to participate in the study and were scheduled for their baseline study visit at 6–9 weeks postpartum. The SWIFT participants provided written consent for 3 in-person study visits at baseline (6–9 weeks postpartum) and annually for 2 years postpartum, and consented to continued clinical surveillance for diagnosis of new onset T2D up to 8.4 years post-baseline (average surveillance period 2.72 years). Quantitative assessments of lactation intensity and duration since delivery were obtained via monthly mailed surveys and for the previous 7 days at in-person research exams. At in-person annual research exams from baseline through 2 years later, participants completed interviewer- and self-administered surveys that gathered data on sociodemographics, medical and reproductive history, family history of diabetes, sleep habits, depression (CES-D), and lifestyle behaviors. Trained research staff also obtained anthropometric measurements (body weight, height, and waist circumference) using calibrated research instruments at each in-person study exam. Clinical data were also obtained from electronic medical records, including laboratory results for the prenatal 3-hour 100-g OGTT used to diagnose gestational diabetes, pre-pregnancy weight, dates of diabetes diagnoses, and other clinical outcomes [32]. At each study visit, plasma samples were collected at the fasting and 2-hour time points during the 75-g OGTT, and the other assessments were completed. The plasma samples were analyzed within several weeks for glucose and insulin levels, and subsequently for selected levels of lipids and lipoproteins, as previously described [33,34], and aliquots were stored in the SWIFT Biobank. Follow-up assessments to determine T2D status were performed via annual study research 2-hour 75-g OGTTs and ongoing review of electronic medical records to capture clinical diagnoses of diabetes from KPNC clinical laboratory tests after baseline [3]. T2D diagnosis was based on ADA criteria [35]. The fasting plasma samples were stored at the KPNC Division of Research Clinic in low-temperature freezers at −80°C. The study design and all procedures were approved by the KPNC Institutional Review Board and the Office of Research Ethics at University of Toronto. All the participants provided written consent for the study.

### Metabolomics

The fasting plasma samples collected from 658 participants at baseline (173 incident T2D cases and 485 controls [pair-matched on BMI, age, and race/ethnicity]) and 337 of the same participants during 2 years of follow-up (98 T2D cases and 239 controls) were evaluated via metabolomics (not all patients delivered follow-up samples). Metabolites associated with T2D were selected based on a literature review of previous T2D metabolic studies. These metabolites were chosen on the basis of consistency in significance in more than 2 studies [1,6,19,25,36–42]. The AbsoluteIDQ p180 plate, covering most of the selected metabolites, was used to assay a total of 188 metabolites using mass-spectrometry-based detection according to manufacturer instructions (Biocrates Life Sciences, Innsbruck, Austria). Aliquoted samples were prepared using the AbsoluteIDQ p180 Kit in accordance with the vendor specifications. In brief, after the addition of 10 μl of the supplied internal standard solution to each well on a filterspot of the 96-well extraction plate, 10 μl of each serum sample, quality control (QC) sample, blank, zero sample, or calibration standard was added to the appropriate wells. Chromatographic separation and measurement of amino acids (AAs) and biogenic amines was performed using an Agilent 1290 HPLC stack connected to a SCIEX QTRAP 5500 mass spectrometer. An Agilent Eclipse XDB-C18 100 × 3.0 mm, 3.5 μm column was used. All AAs (21), biogenic amines (21), and hexose utilize either deuterated or 13C stable isotope-labeled internal standard of the exact analyte or a closely eluting compound of similar class and were analyzed by LC-MS/MS. Acylcarnitines (40), sphingolipids (15), and glycerophospholipids (90) were analyzed by flow injection analysis tandem mass spectrometry (FIA-MS/MS) and quantified by internal standard calibration using an Agilent 1200 HPLC stack connected to a SCIEX QTRAP 5500 mass spectrometer. Multiple reaction monitoring (MRM) transitions for each analyte and internal standard were collected over a scheduled retention time window using tune files and acquisition methods provided in the AbsoluteIDQ p180 Kit. The data were then imported to the Biocrates software MetIDQ, which validates the plate and calculates the concentration values. The median value of all zero samples on the plate was calculated as an approximation of background noise, i.e., as the limit of detection (LOD). All assays were performed and assessed, without disclosure of group allocation, by the Analytical Facility for Bioactive Molecules (The Hospital for Sick Children, Toronto, ON, Canada).

### Prediction analysis

Analytes with >10% of measurements below LOD were excluded from prediction analysis. Out of 188 analytes, 132 were subjected to prediction analysis. For these 132 analytes, samples with values reported as “<LOD” were imputed using the LOD/2 value for each specific analyte. To build and evaluate the prediction model, we randomly selected 51 future T2D cases and 51 non-T2D controls as hold-out testing set. The rest of the participants (122 future T2D cases and 434 non-T2D controls) were used as a training set. The training set was randomly down-sampled to a case–control balanced set (122 cases versus 122 controls) for generating prediction models using random forest classification (package randomForest in R program). The model was further applied to the hold-out testing set to evaluate the prediction performance. The process of model generation and evaluation was repeated 100 times (S1A Fig). The top 30 variable importance (VIP) analytes were recorded at each time. The prediction ability of each analyte was ranked by the frequency of its appearance in the 100 times’ top 30 VIP lists. The 20 analytes with the highest ranking, demonstrating the best prediction performance, were selected as the signature panel. The signature panel was used for model generation and evaluation following the process in S1A Fig again. Evaluation results in hold-out testing sets, including sensitivity, specificity, precision, F1-score, AUC, and accuracy, were calculated. Instead of recording the best model, we reported the performance of all the models in their hold-out test sets from the 100 repetitions to avoid the potential overfitting and bias. All the analyses were done in the open-source R program version 3.2.4.

### Metabolomics data analysis in baseline and follow-up cross-sectional studies

Analytes with >40% of measurements below LOD were excluded from the metabolomics data analysis, only allowing the most robust analytes from the panel for the analysis; this reduced the total number of analytes reported in the dataset from 188 analytes to 141 analytes (baseline data) and 145 analytes (follow-up data) [43]. For these 141 and 145 analytes, samples with values reported as “<LOD” were imputed using the LOD/2 value for each specific analyte. Log-transformation was performed. Generalized linear models (GLMs) were fit, and Type III ANOVA tests were performed in SPSS Statistics version 25 (IBM, Armonk, NY) to identify analytes that differ between the future T2D cases and non-T2D controls, adjusting for effects from race/ethnicity, age, and BMI: log2analytes ~ group (disease or not) + BMI + race/ethnicity + age. The false discovery rate (FDR) was acquired by correcting the *p*-value by the Benjamini–Hochberg method for multiple comparison, and a cutoff of 0.05 was used for baseline data analyses. Pearson correlation was used to assess the correlation between metabolites and clinical parameters at baseline. Correlation coefficients (*r*) and *p*-value were calculated.

### Pathway analysis

The differentially expressed metabolites with their Human Metabolome Database (HMDB) accession numbers were subjected to pathway analysis using the Kyoto Encyclopedia of Genes and Genomes (KEGG; Kanehisa Laboratories, Kyoto, Japan) pathway database, and enrichment analysis using the Small Molecule Pathway Database (SMPDB). Pathway analyses were carried out on platform MetaboAnalyst 4.0.

### Longitudinal analysis

Metabolomics data from 337 samples (98 progressors pair-matched with 239 non-progressors for age, race/ethnicity, and BMI) measured both at baseline and follow-up were processed. Individual metabolites with >40% missing values (measurements < LOD) either in baseline or follow-up data were excluded at both time points, which reduced the total analytes in the dataset from 188 to 140. Batch correction was performed on baseline and follow-up data according to the same internal control detected in 2 batches. Log-transformation and normality test were performed. A mixed effect model was fitted for each individual analyte, and Type III ANOVA tests were performed in SPSS: log2analytes ~ group (progressor versus non-progressor) + time point (baseline or follow-up) +group × time point + patient ID. Group effects, time-dependent effects, and their interactions were included as fixed effects, and patient ID was included as a random effect. The resulting *p*-values were corrected using the Benjamini–Hochberg method, and a cutoff of 0.2 was used for the significance.

This study is reported as per the Strengthening the Reporting of Observational Studies in Epidemiology (STROBE) guideline (S2 Text).

## Results

### Study cohort overview

**Fig 1:**
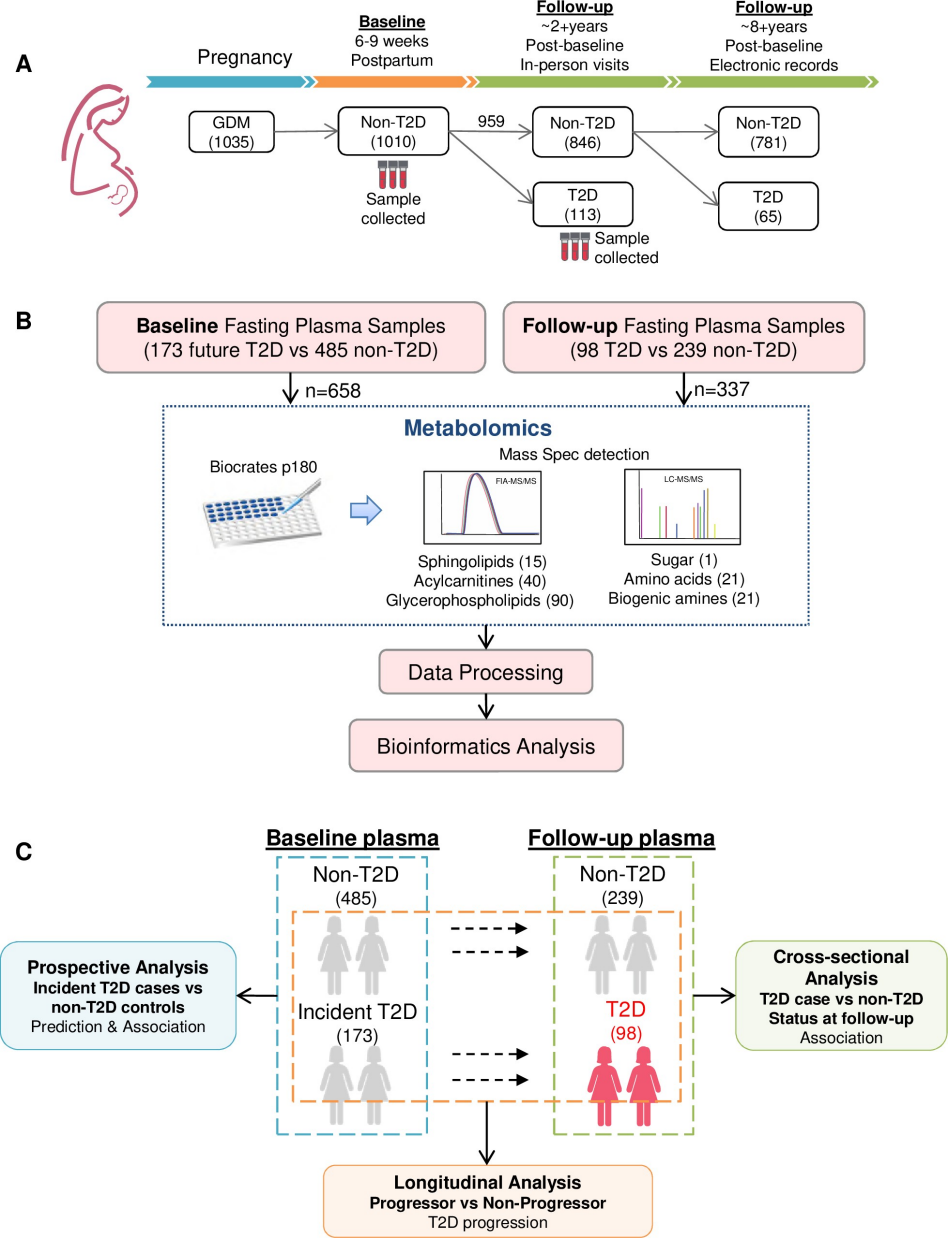
The Study of Women, Infant Feeding and Type 2 Diabetes after GDM Pregnancy (SWIFT) cohort and study design. (A) SWIFT prospective cohort: 1,035 women diagnosed with gestational diabetes mellitus (GDM) in 2008–2011 were enrolled at 6–9 weeks postpartum (baseline). Of the 1,035 participants, 1,010 were confirmed via 2-hour 75-g oral glucose tolerance test to be without diabetes at baseline, underwent annual 2-hour 75-g OGTTs for 2 years post-baseline, and subsequently had their electronic medical records searched for clinical diagnoses of diabetes. Up to 8 years post-baseline, a total of 178 (18%) women developed type 2 diabetes (T2D) after baseline. Out of those 178 incident cases, 113 women were diagnosed as having diabetes clinically during the 2 years of in-person follow-up (blood samples of 98 women were available at this time point). (B) Metabolomics: using Biocrates p180 kits, 188 metabolites were measured in fasting plasma samples from participants at baseline (173 incident T2D cases and 485 nested pair-matched non-T2D controls) and follow-up (98 T2D cases and 239 nested pair-matched non-T2D controls). (C) The bioinformatics analysis pipeline includes 3 integral parts: (1) prospective analysis to predict future diabetes and to profile metabolic changes associated with future T2D onset at baseline, (2) cross-sectional analysis of the follow-up samples to reveal T2D-associated metabolic pathways, and (3) longitudinal analysis to shape a trajectory of T2D progression.

In the SWIFT cohort, a total of 1,035 women diagnosed with GDM were recruited during gestation and enrolled into the study at 6 to 9 weeks postpartum (study baseline). Of these, 1,010 women were found to be without T2D based on the results of a 2-hour 75-g OGTT at baseline. Among the 1,010 women, 959 subsequently completed annual in-person study visits including research 2-hour OGTTs up to 2 years post-baseline (95% cohort retention) to reclassify glucose tolerance, and/or had additional testing of glycemia for clinical diagnosis of new onset T2D in KPNC electronic health records within 8 years post-baseline. Fasting plasma samples were collected at SWIFT study baseline (2008 to 2011) and at each in-person follow-up study visit at 1 year and 2 years post-baseline (2009 to 2014). As of December 2017, among the 959 women without T2D at baseline and with follow-up testing, 178 women (18%) had developed incident T2D after baseline (Fig 1A). In our nested case–control study design, we selected pair-matched participants based on diabetes status and time of follow-up, with 173 incident T2D cases (5 baseline samples were not available to be measured) and 485 non-T2D controls (658 women in total) who were profiled for metabolomics in fasting plasma samples previously obtained at 6–9 weeks postpartum (baseline). Further, fasting plasma samples from the follow-up visits in the same cohort underwent metabolomics analysis (98 T2D cases and 239 nested pair-matched non-T2D controls) (Fig 1A and 1B). The data analysis was composed of 3 integral parts: (1) differentiation of baseline metabolite profiles for the subsequent incident T2D cases and non-T2D controls, to develop a predictive model for future new onset T2D and to delineate metabolic changes associated with the transition from GDM to T2D; (2) cross-sectional analysis of the follow-up samples concurrent with diabetes case–control status to reveal T2D-associated metabolic pathways; and (3) longitudinal analysis to shape and present a trajectory of T2D progression by tracing metabolic changes within individuals (Fig 1C).

### Cohort clinical characteristics

Sociodemographic and clinical parameters at baseline are summarized in Table 1. For prenatal characteristics, there was no significant difference in age, race/ethnicity, or parity between women who later developed T2D and those who did not. Pre-pregnancy BMI (*p <* 0.001) and prenatal 3-hour 100-g OGTT (sum of the 4 *z*-scores for glucose values; fasting and 1 hour, 2 hours, and 3 hours post-load, *p <* 0.001) for the incident T2D case group were higher than those in the matched non-T2D control group, similar to those in the entire cohort of 959 women with the 1- or 2-year follow-up glycemic testing [3]. Compared to the non-T2D control group, a higher percentage of participants in the incident T2D case group had a family history of diabetes (*p =* 0.018) and had been treated with insulin or oral medications during pregnancy (*p <* 0.001). At study baseline, compared to controls, women in the incident T2D group had higher BMI (*p =* 0.02), FPG, 2-hour plasma glucose (2hPG), fasting insulin, and 2-hour insulin (all *p*-values < 0.001), and higher fasting triacylglycerol (TAG) (*p =* 0.005), fasting ln triglycerides (*p =* 0.002), homeostatic model assessment for insulin resistance (HOMA-IR) (*p <* 0.001), and percentage of kilocalories as animal fat in dietary intake (*p =* 0.006), but lower fasting high-density lipoprotein cholesterol (HDL-C) (*p =* 0.008) and low-density lipoprotein cholesterol (LDL-C) (*p =* 0.044). There was no significant difference in fasting total cholesterol, homeostatic model assessment for beta cell function (HOMA-B), hypertension percentage, smoker percentage, dietary glycemic index, and physical activity score at baseline between incident T2D case and non-T2D control groups.

**Table 1:** Prenatal and study baseline (6–9 weeks postpartum) characteristics of women with gestational diabetes mellitus in the SWIFT cohort.

Characteristic	Incident T2D (*n* = 173)	Non-T2D (*n* = 485)	*p*-Value
**Prenatal characteristics**			
Age (years), mean (SD)	33.2 (5.2)	32.9 (4.5)	0.53
Race/ethnicity, *n* (%)			0.98
White	29 (16.7)	89 (18.4)	
Asian	52 (29.9)	144 (29.8)	
Black	22 (12.6)	55 (11.4)	
Hispanic	69 (39.7)	190 (39.3)	
Other	2 (1.2)	6 (1.24)	
Parity, *n* (%)			0.92
Primiparous (1 birth)	55 (31.6)	161 (33.3)	
Biparous (2 births)	65 (37.4)	177 (36.6)	
Multiparous (>2 births)	54 (31.0)	146 (30.2)	
GDM treatment, *n* (%)			<0.001
Diet only	75 (43.1)	346 (71.5)	
Oral medications	80 (46.0)	130 (26.9)	
Insulin	19 (10.9)	8 (1.7)	
Pre-pregnancy BMI (kg/m^2^), mean (SD)	33.6 (8.3)	31.6 (6.7)	<0.001
Sum of prenatal 3-hour 100-g OGTT glucose *z*-scores, mean (SD)	1.3 (3.1)	−0.2 (2.5)	<0.001
Family history of diabetes, *n* (%)	104 (59.8)	239 (49.4)	0.018
**Baseline characteristics at 6–9 weeks postpartum**			
BMI (kg/m^2^), mean (SD)	33.4 (7.6)	31.9 (6.4)	0.02
Fasting plasma glucose, mmol/l, mean (SD)	5.6 (0.6)	5.2 (0.4)	<0.001
2-hour post-load plasma glucose (75-g OGTT), mmol/l, mean (SD)	7.3 (1.6)	6.0 (1.4)	<0.001
Fasting plasma insulin pmol/l, median (IQR)	181.3 (124.3–265.3)	139.6 (96.5–207.7)	<0.001
2-hour plasma insulin, pmol/l, median (IQR)	771.6 (502.1–1,074.4)	565.3 (375.0–822.3)	<0.001
Fasting plasma triglycerides, mmol/l, median (IQR)	1.3 (0.9–2.1)	1.1 (0.8–1.7)	0.005
Fasting plasma ln triglycerides, mmol/l, mean (SD)	0.34 (0.6)	0.19 (0.5)	0.002
Fasting plasma HDL-C, mmol/l, mean (SD)	1.26 (0.3)	1.33 (0.4)	0.008
Fasting plasma total cholesterol, mmol/l, mean (SD)	5.1 (0.9)	5.2 (0.9)	0.19
Fasting plasma LDL-C, mmol/l, mean (SD)	3.1 (0.8)	3.3 (0.8)	0.04
HOMA-IR, median (IQR)	6.6 (4.5–10.1)	4.6 (3.2–7.1)	<0.001
HOMA-B, median (IQR)	260 (172–366)	240 (181–345)	0.33
Hypertension, *n* (%)	13 (7.5)	25 (5.2)	0.30
Smoker, *n* (%)	5 (2.9)	9 (1.9)	0.43
Dietary glycemic index, mean (SD)	244.7 (107.7)	238.1 (107.1)	0.49
Dietary intake, percentage of kilocalories as animal fat, mean (SD)	27.0 (7.6)	25.0 (8.5)	0.006
Physical activity score, met-hours per week, mean (SD)	51.3 (24.0)	47.3 (20.7)	0.06

Data are presented as the mean (SD) unless otherwise noted.

Chi-square test for categorical variables (n, %), t-test for continuous variables (Mean, SD), and Wilcoxon rank-sum test (Median, IQR).

GDM, gestational diabetes mellitus; HDL-C, high-density lipoprotein cholesterol; HOMA-B, homeostatic model assessment for beta cell function; HOMA-IR, homeostatic model assessment for insulin resistance; LDL-C, low-density lipoprotein cholesterol; OGTT, oral glucose tolerance test; T2D, type 2 diabetes.

### Developing a metabolic signature predicting future T2D

**Fig 2:**
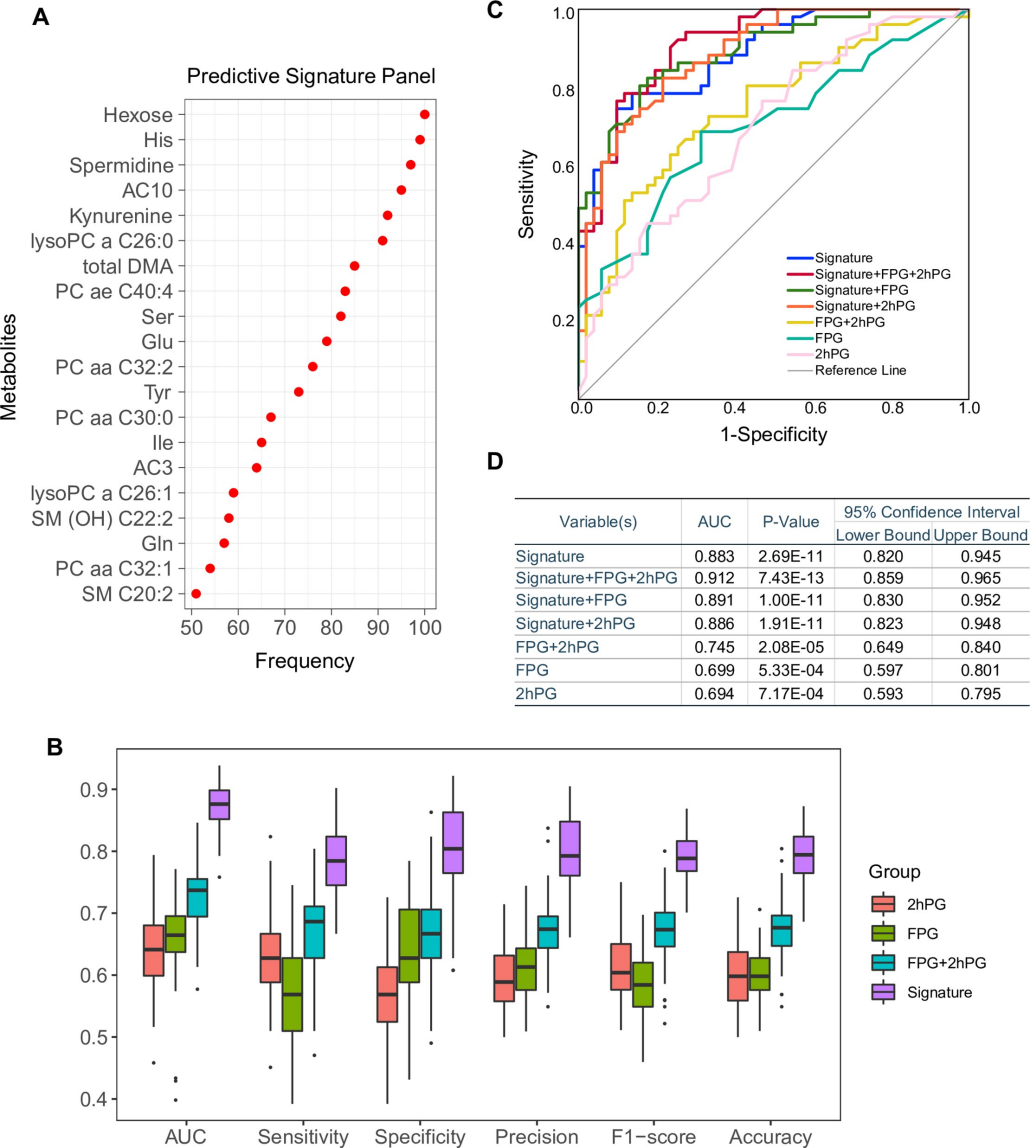
Metabolic signature and models to predict future type 2 diabetes. (A) Random forest variable selection identified a set of 20 metabolites with high predictive power. (B) Box plots showing the distribution of the predictive performance of the metabolic signature and the traditional clinical parameters 2-hour plasma glucose (2hPG) and fasting plasma glucose (FPG). (C) Receiver operating characteristic (ROC) curve of predictive models generated by metabolic signature and clinical parameters. (D) Area under the ROC curve (AUC) and its 95% CI, with significance indicated by *p*-value.

A signature composed of 20 metabolites—hexose, 6 AAs, 6 glycerophospholipids, 2 acylcarnitines, 2 sphingolipids, and 3 biogenic amines—was developed (Fig 2A). A prediction model was generated in each randomly sampled training set using this 20-metabolite signature. The model was applied to the corresponding hold-out testing set for validation, and its predictive performance was evaluated by the AUC, sensitivity, specificity, precision, F1-score, and accuracy. This process of model generation and evaluation was repeated 100 times (S1A Fig). The routine clinical parameters FPG and 2hPG were also used for prediction analysis using the same process. Using the 20-metabolite signature, we achieved the predictive performance median ± SD values AUC 0.88 ± 0.03, sensitivity 0.78 ± 0.05, specificity 0.80 ± 0.07, precision 0.79 ± 0.06, F1-score 0.79 ± 0.04, and accuracy 0.79 ± 0.04, superior to the traditional clinical parameters FPG and 2hPG or their combination (Figs 2B and S1B). To avoid any preferential bias, we chose the training/testing partition with median performance to present the receiver operating characteristic curve for comparison of models built upon variant parameters (Fig 2C). Using the signature, we achieved AUC 0.883 (95% CI 0.820–0.945, *p* < 0.001), which is better than FPG (AUC 0.699, 95% CI 0.597–0.801, *p* < 0.001), 2hPG (AUC 0.694, 95% CI 0.593–0.795, *p* < 0.001), and their combination (AUC 0.745, 95% CI 0.649–0.840, *p* < 0.001). Interestingly, when clinical parameters (FPG or 2hPG) were added to the 20-metabolite signature panel, discrimination was not improved using either of the clinical parameters, and only slightly improved using both (Fig 2D), indicating the significance of using the metabolite signature to predict diabetes. Taken together, we developed a signature panel predicting future T2D with AUC 0.88 ± 0.03. This suggests that metabolic changes occur before T2D onset, which allows us to predict diabetes and further explore the metabolic changes associated with T2D incidence.

### Prospective analysis at baseline: Metabolic changes associated with future T2D

**Fig 3:**
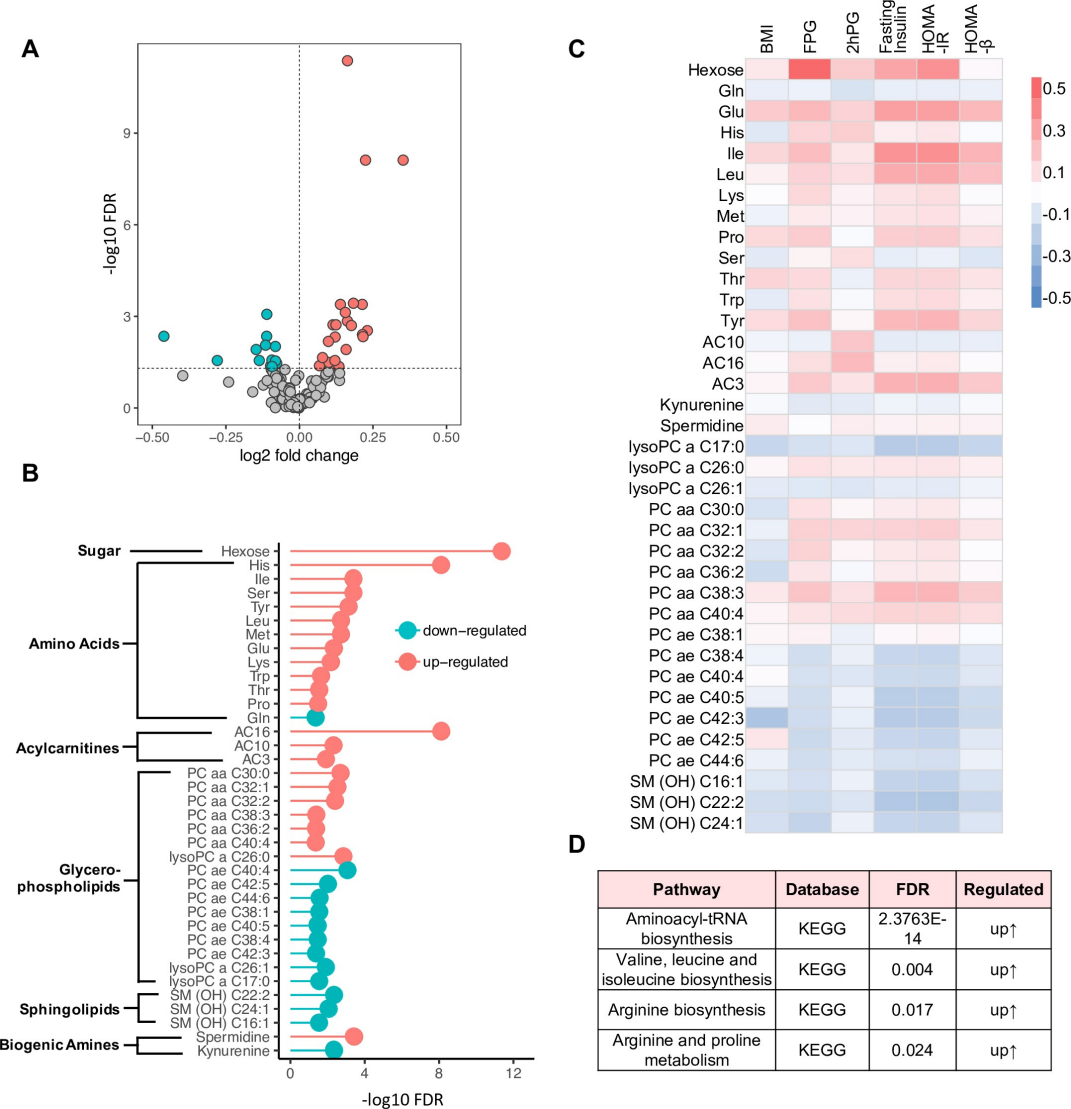
Metabolites and pathways associated with future type 2 diabetes (T2D) at baseline. (A) Volcano plot shows -log10 FDR against log2-fold change of 141 metabolites in incident T2D group versus non-T2D controls. Green denotes down-regulated metabolites, red denotes up-regulated metabolites, and grey denotes no change. (B) 37 differentially regulated metabolites were associated with incidence of future T2D. Red indicates up-regulation, and green indicates down-regulation. Significance is shown as -log10 FDR. (C) Correlation analysis of the 37 differential metabolites with clinical parameters at baseline using Pearson method. Red boxes indicate positive correlation coefficient (*r*), and blue boxes, negative. (D) Biological pathways associated with future diabetes onset. 2hPG, 2-hour plasma glucose; FDR, false discovery rate; FPG, fasting plasma glucose; HOMA-B, homeostatic model assessment for beta cell function; HOMA-IR, homeostatic model assessment for insulin resistance; KEGG, Kyoto Encyclopedia of Genes and Genomes.

Of 188 metabolites measured, 141 passed data preprocessing and were subjected to bioinformatics analysis. Partial least squares discriminant analysis (PLS-DA) indicated separability between the future T2D cases and non-T2D controls, and differential metabolic profiles in the 2 groups before disease onset (S2A–S2C Fig). To further delineate the differentially expressed metabolites between incident T2D case and non-T2D control samples, individual analytes were subjected to linear regression models, and an ANOVA was performed to evaluate significance [44]. These models were adjusted for race/ethnicity, age, and BMI. Thirty-seven metabolites were identified that were differentially expressed between incident T2D cases and non-T2D controls with statistical significance (FDR < 0.05), including 23 up-regulated and 14 down-regulated metabolites (Fig 3A and 3B; S1 Table). The abundance of metabolites in individual participants is presented in the heat map shown in S2D Fig. Hexose was reported to be increased in incident T2D cases by us and others [19,37,38]. In the current study, at baseline, hexose was the most significantly increased metabolite (FDR < 0.001), with the non-T2D group at 5.45 ± 0.8 mM and the incident T2D case group at 6.10 ± 0.1 mM (S1 Table). This suggests that hexose metabolism is the most regulated, and the most strongly associated with disease onset.

AA metabolism was also altered between incident T2D cases and non-T2D controls. Eleven AAs (histidine, isoleucine, serine, tyrosine, leucine, methionine, glutamate, lysine, tryptophan, threonine, and proline) were up-regulated, with only glutamine being down-regulated. Acylcarnitines and biogenic amines (related to AA metabolism) were also affected. Specifically, increases of the acylcarnitines AC3 (FDR = 0.01), AC10 (FDR = 0.005), and AC16 (FDR < 0.001) were observed before the occurrence of T2D. Spermidine, a biogenic amine, was found to be increased, while another type of amine, kynurenine, was decreased. In addition to these metabolites, sphingomyelins were also found to be decreased, which is consistent with the report in our previous study [19]. Similarly, lysophosphatidylcholines (lysoPCs) were also decreased except for lysoPC26:0. Interestingly, analytes from the glycerophospholipids group were detected in both up-regulated and down-regulated sets. The up-regulated glycerophospholipids were of the PC aa C group (diacyl-glycerophospholipids), and the down-regulated glycerophospholipids were of the PC ae C group (acyl-alkyl-glycerophospholipids).

The correlations of the 37 differentially expressed metabolites and clinical parameters (BMI, FPG, 2hPG, fasting insulin, HOMA-IR, and HOMA-B) at study baseline before transition to T2D are presented in Fig 3C and S2 Table. As expected, hexose was shown to have the highest correlation with FPG (*r =* 0.58, *p <* 0.001). It also correlated with fasting insulin (*r =* 0.30, *p* < 0.001) and HOMA-IR (*r =* 0.36, *p <* 0.001) but not HOMA-B (*r =* 0.01, *p =* 0.71). In contrast, relatively low correlations between metabolites and BMI or 2hPG (−0.20 < *r* < 0.20) were detected. The majority of AAs (and derivatives) and the PC aa C group were shown to positively correlate with fasting insulin, FPG, and HOMA-IR, while the PC ae C group and sphingolipids were shown to have negative correlations. BCAAs (leucine and isoleucine), glutamate, and tyrosine were positively correlated with fasting insulin (Leu, *r =* 0.28, *p <* 0.001; Ile, *r =* 0.36, *p <* 0.001; Glu, *r =* 0.31, *p <* 0.001; Tyr, *r =* 0.23, *p* < 0.001) and HOMA-IR (Leu, *r =* 0.28, *p* < 0.001; Ile, *r =* 0.37, *p <* 0.001; Glu, *r =* 0.32, *p <* 0.001; Tyr, *r =* 0.24, *p* < 0.001). Acylcarnitine AC3 was also positively correlated with fasting insulin (*r =* 0.25, *p* < 0.001) and HOMA-IR (*r =* 0.26, *p* < 0.001).

To further investigate the molecular pathways underlying T2D onset and identify affected biological pathways, we used KEGG for pathway analysis. The aminoacyl-tRNA biosynthesis pathway (FDR < 0.001) as well as BCAA biosynthesis (FDR = 0.004) and arginine biosynthesis (FDR = 0.017) from KEGG were found to be up-regulated in women who transitioned to T2D during follow-up compared to non-T2D controls (Fig 3D; S3 Table). Interestingly, arginine/proline metabolism (FDR = 0.024) was also up-regulated (Fig 3D; S3 Table). These findings suggest hyperactive AA metabolism might play an important role in future diabetes onset.

### Cross-sectional analysis at follow-up: Metabolic changes associated with T2D

**Fig 4:**
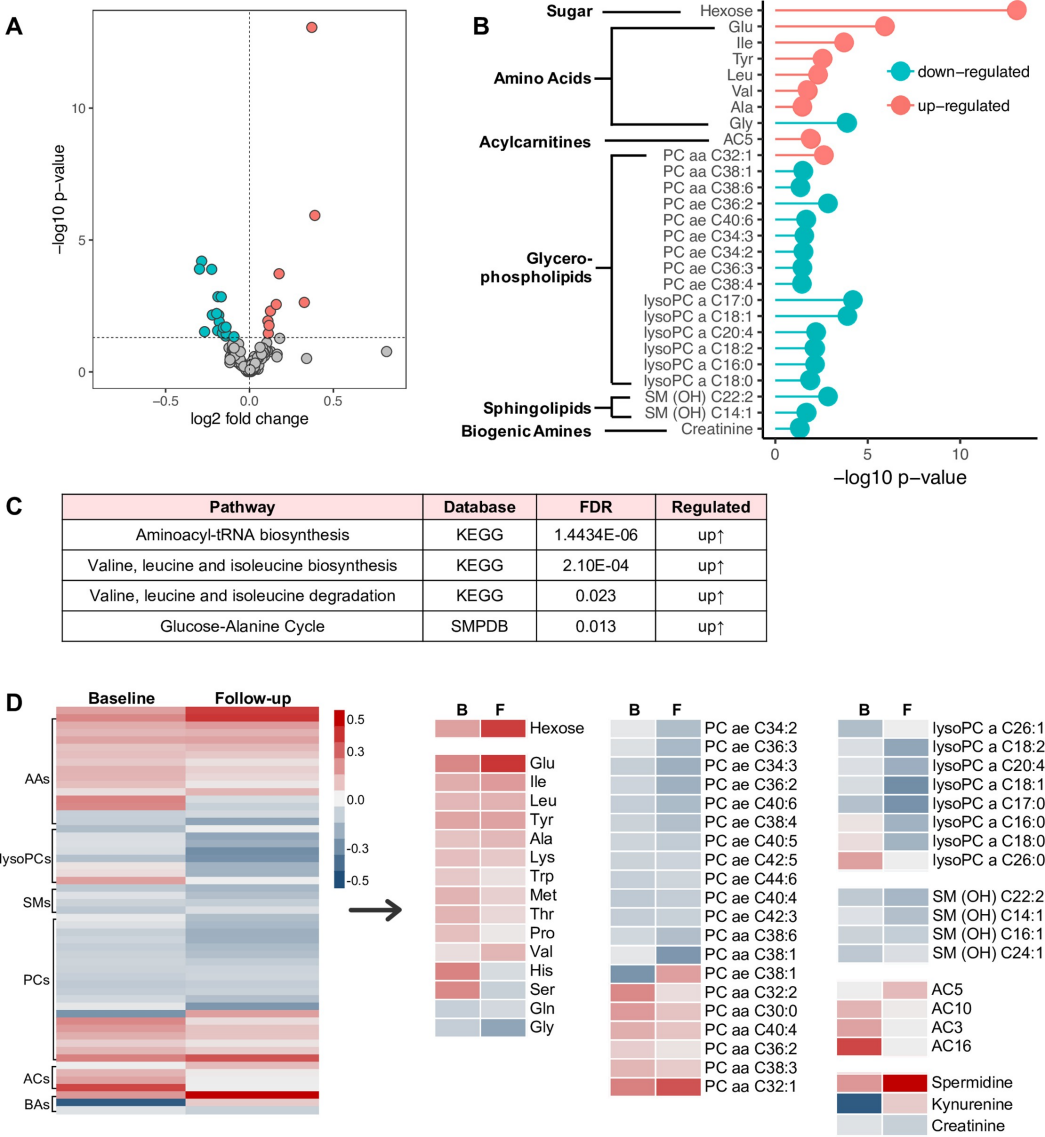
Metabolites associated with type 2 diabetes (T2D) at follow-up. (A) Volcano plot showing −log10 *p*-value against log2-fold change of 145 metabolites in T2D cases versus non-T2D controls. Plotted are down-regulated (green), up-regulated (red), and unchanged (grey) metabolites. (B) 27 differentially regulated metabolites were associated with T2D. Significance is shown as −log10 *p*-value. (C) Regulated pathways associated with T2D at follow-up. (D) Comparison of the differential metabolites between baseline (B) and follow-up (F). Fold changes are log transformed and indicated by color scale in matrix. AAs, amino acids; ACs, acylcarnitines; BAs, biogenic amines; KEGG, Kyoto Encyclopedia of Genes and Genomes; lysoPCs, lysophosphatidylcholines; PCs, glycerophospholipids; SMPDB, Small Molecule Pathway Database; SMs, sphingolipids.

Follow-up samples (98 T2D cases out of 173 provided follow-up samples, while 239 pair-matched non-T2D controls out of 485 provided follow-up samples) were further analyzed cross-sectionally for identification of T2D-associated metabolites. Similar to what was observed at baseline, T2D participants were separated from non-T2D participants based upon differentially expressed metabolic profiles in the PLS-DA score plot, which suggested significant metabolic changes in T2D compared to non-T2D participants (S3A–S3C Fig). In particular, a total of 27 differentially regulated metabolites were identified in the T2D group compared to the non-T2D group with statistical significance (*p <* 0.05), including 9 up-regulated and 18 down-regulated metabolites (Fig 4A and 4B; S4 Table). Predictably, hexose, presumably primarily glucose, was increased, with the highest significance (*p* < 0.001). This is consistent with the clinical diagnosis of T2D. Notably, in all of 27 differentially regulated metabolites, a group of 6 AAs (glutamate, isoleucine, tyrosine, leucine, valine, and alanine), PC aa C32:1, and AC5 were elevated in the T2D group compared to the non-T2D group (Fig 4A and 4B; S4 Table). In contrast, another 14 glycerophospholipids, 1 AA (glycine), 1 biogenic amine (creatinine), and 2 sphingolipids [SM(OH)C22:2 and SM(OH)C14:1] were decreased in T2D versus non-T2D participants (Fig 4A and 4B; S4 Table). These regulated metabolites, including carbohydrates (glucose and fructose), lipids (phospholipids and sphingomyelins), and AAs (BCAAs, aromatic AAs, and glycine), were reported previously in T2D cohorts (general adult population) [38]. Metabolite abundance in individuals demonstrated variance between participants (S3D Fig).

To gain further insight into the metabolic changes associated with T2D, a KEGG pathway analysis of all differential metabolites was performed. Similar to our findings at baseline, KEGG pathway analysis revealed that AA metabolism was profoundly affected. The aminoacyl-tRNA biosynthesis pathway was most enriched in participants with T2D (FDR < 0.001), along with BCAA biosynthesis and degradation pathways from KEGG (FDR < 0.001 and FDR = 0.023, respectively) (Fig 4C; S5 Table). More importantly, the glucose-alanine cycle pathway, in which nitrogen goes into the urea cycle and glucose is made, was up-regulated (FDR = 0.013) (Fig 4C; S5 Table).

Further, all the differentially regulated metabolites were compared between baseline and follow-up in Fig 4D. Hexose, which was elevated at baseline showed a greater increase at follow-up (increase by 15%). Most of the AAs maintained the same pattern (increased or decreased) except for histidine and serine, which were increased at baseline but decreased at follow-up (Fig 4D). The majority of phospholipids (lysoPCs and the PC ae C group, except for lysoPC a 26:0, 16:0, and 18:0 and PC ae C38:1) and sphingolipids maintained a decreasing pattern at both time points (Fig 4D). In contrast, phospholipids of the PC aa C group (except for PC aa C38:1 and C38:6) maintained an upward pattern at both time points. Spermidine and creatinine maintained their patterns at both time points, while kynurenine was decreased at baseline but increased at follow-up (Fig 4D). The elevated AC3, AC10, and AC16 at baseline were normalized at follow-up, but AC5 was increased (Fig 4D). Most differentially regulated metabolites identified at baseline maintained their changes at follow-up.

### Longitudinal analysis: Metabolites in individuals associated with the transition from GDM to T2D

**Fig 5:**
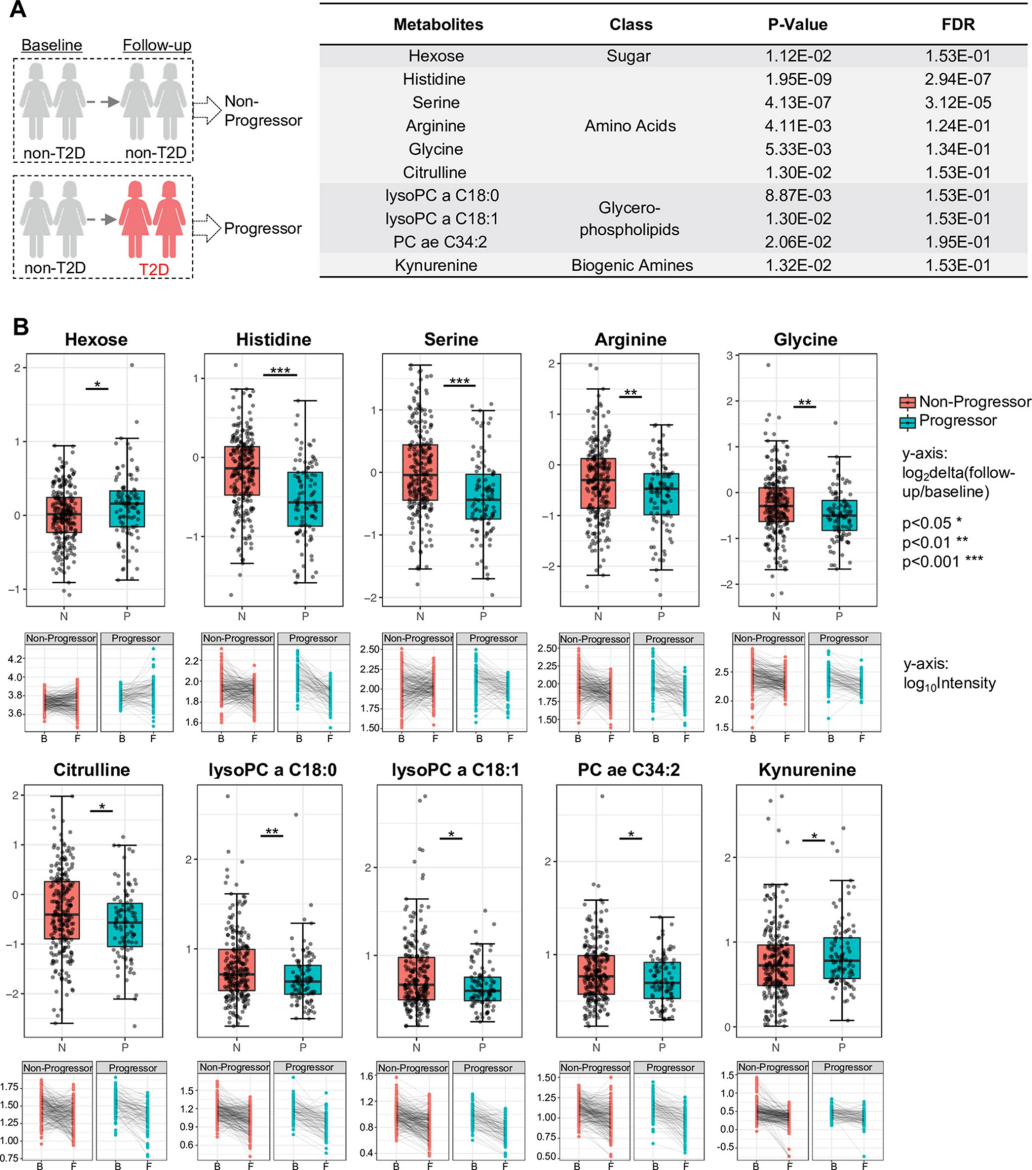
Metabolites associated with type 2 diabetes (T2D) progression in longitudinal study. (A) 10 Metabolites significantly differ in progressors versus non-progressors across T2D development. *p*-Values < 0.05; *p*-values were obtained by Type III ANOVA test in a mixed effect model. False discovery rate (FDR) values < 0.2; FDR values were obtained by correcting *p*-value by Benjamini–Hochberg method. (B) Delta values (upper panels) and trajectory (lower panels) of 10 metabolites within all individuals during diabetes progression.

To further examine metabolite dynamics and illuminate a metabolic path preceding T2D onset, the dynamic changes of metabolites within each individual were traced. In this longitudinal analysis, we selected 337 participants who had been analyzed both at baseline and follow-up. A total of 98 participants developed T2D during follow-up and were defined as progressors, whereas 239 did not develop T2D, defined as non-progressors (Fig 5A). By using multiple linear regression models, we identified 10 metabolites that significantly changed over the time course between progressors and non-progressors (*p <* 0.05, FDR < 0.2). These 10 metabolites—hexose, 5 AAs, 1 biogenic amine, and 3 phospholipids—link metabolic changes to diabetes progression (Fig 5A). The relative concentration change in each metabolite within each individual was traced, which led to the formation of a trajectory for each metabolite over time (Fig 5B). Specifically, delta values of hexose and kynurenine were elevated during the period for diabetes progression, whereas delta values of the remaining 8 metabolites (histidine, serine, arginine, citrulline, glycine, lysoPC a C18:0, lysoPC a C18:1, and PC ae C34:2) were decreased (Fig 5B). As shown in longitudinal analysis, these 10 metabolites with changes of delta values were suggested to contribute to T2D progression (Figs 5B and S4). Far fewer differentially dysregulated metabolites were identified here compared to in the cross-sectional analysis (baseline and follow-up), suggesting most metabolic changes at baseline were maintained throughout the T2D progression.

### Building a metabolic profile for the transition from GDM to T2D: Integrating baseline/follow-up cross-sectional and longitudinal analyses

**Fig 6:**
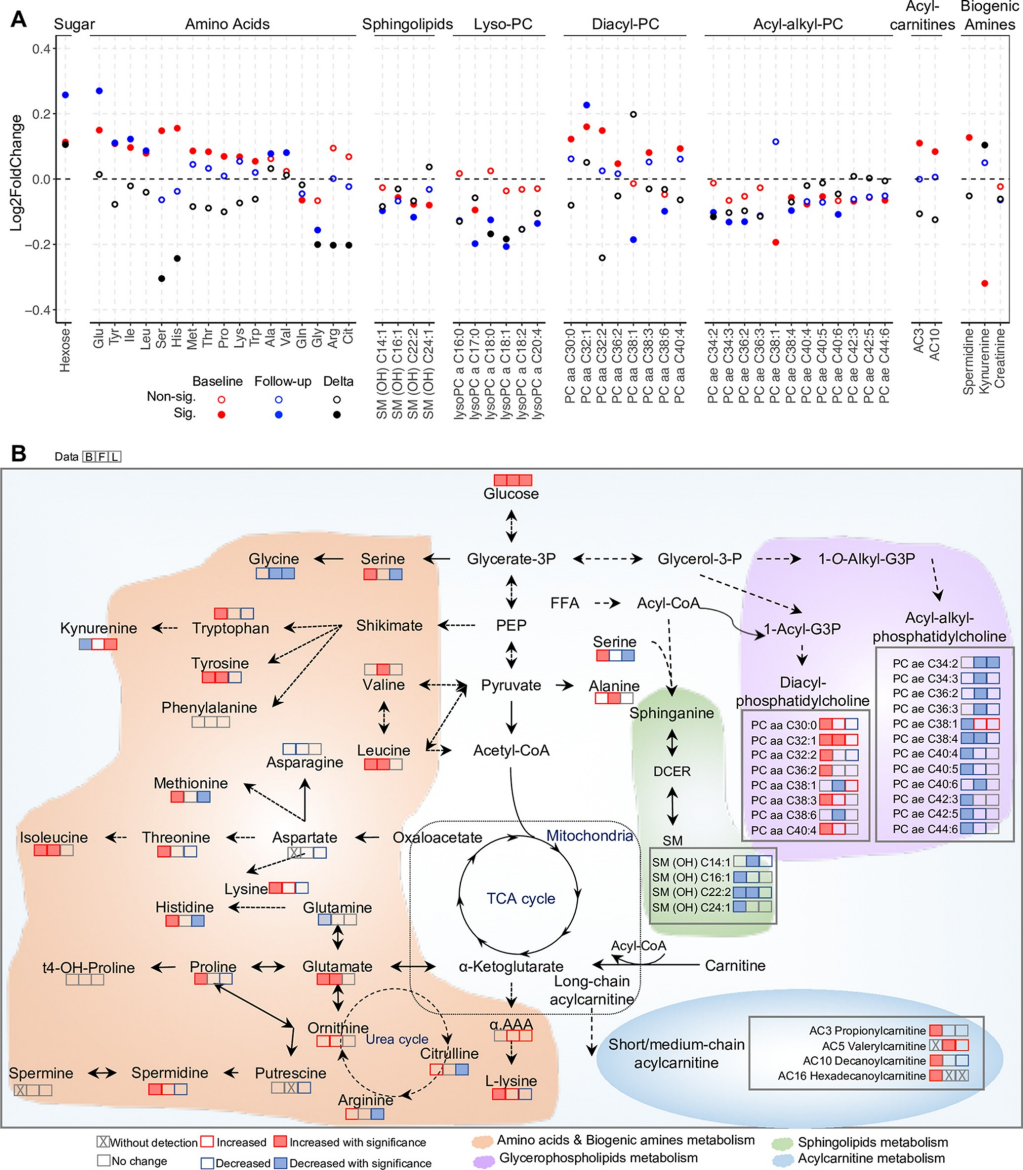
A putative model depicts metabolic changes during the transition from gestational diabetes mellitus (GDM) to type 2 diabetes (T2D). (A) An integrative metabolic profile of diabetes onset and progression from meta-analysis of baseline prospective, follow-up cross-sectional, and longitudinal studies. Red, blue, and black dots denote metabolite changes in baseline, follow-up, and longitudinal (delta) data, respectively. Solid dots represent significance, empty dots non-significance. (B) The integrated metabolic networks of amino acid, acylcarnitine, and lipid dysmetabolism in the transition from GDM to T2D. Triplets of squares denote baseline (B), follow-up (F), and longitudinal (L) study data, respectively. Solid squares in red and blue represent significant changes, empty squares denote non-significance. Fold change value between 0.95 and 1.05 was considered no change.

All the differentially expressed metabolites detected in baseline/follow-up cross-sectional and longitudinal analyses were selected to build an integrative metabolic profile during transition from GDM to new onset T2D (Fig 6A). At both baseline and follow-up, hexose, the majority of AAs (including BCAAs and aromatic AAs), acylcarnitines (AC3 and AC10), biogenic amines (spermidine), and diacyl-phosphatidylcholines were increased in T2D (Fig 6A). By contrast, the levels of glutamine, glycine, sphingolipids, lysoPCs, and acyl-alkyl-phosphatidylcholines are negatively associated with T2D (Fig 6A). Distinct from baseline and follow-up cross-sectional studies, only 10 metabolites were differentially regulated in the longitudinal study. The longitudinal analysis revealed that the delta values of hexose and the biogenic amine kynurenine increased, but histidine, serine, glycine, arginine, and citrulline decreased, over the transition period (Fig 6A). The delta values of phospholipids lysoPC a C18:0, lysoPC a C18:1, and PC ae C34:2 also decreased. Analytes in T2D case individuals that were not statistically changed in the longitudinal analysis must have been different between controls and cases at baseline (Fig 6A). This suggests that a metabolic dysmetabolism exists at baseline (postpartum) in women with GDM who progress to T2D, including metabolic networks of carbohydrates, AAs, acylcarnitines, and lipids (Fig 6B).

## Discussion

In the present study, targeted metabolomics employing a panel of analytes, many of which had previously been associated with diabetes risk [1,6,19,25,36–42], was used to assess metabolic changes in a well-characterized and racially and ethnically diverse prospective cohort of women with GDM who were tested in the early postpartum period (6 to 9 weeks) and followed up for up to 8.4 years for future onset of T2D (12% developed T2D within 2 years). We discovered a distinct metabolic signature in the early postpartum period that predicted future T2D with predictive power AUC 0.883 (95% CI 0.820–0.945, *p* < 0.001). To further explicate the pathophysiology of the transition from GDM to T2D, we performed both cross-sectional and longitudinal analyses. At baseline, the most striking finding was an upregulated AA metabolism, arginine/proline metabolism, and BCAA metabolism. At follow-up, the up-regulation of AAs was sustained or strengthened in those who developed T2D. Longitudinal analyses revealed only 10 metabolites that were associated with progression to T2D, implicating AA metabolism.

The present study provides insight into the potential utility of a metabolic signature in the early diagnosis of T2D in women with previous GDM. Employing machine learning, we identified 20 metabolites (spanning 3 major metabolite groups including carbohydrates, proteins, and lipids) predicting the transition to incident T2D after GDM. We performed an additional analysis taking into account disease development time from baseline using Cox regression. Comparing this to the logistic regression model, we found that changed metabolites from both models largely overlapped (S6 Table), suggesting variance in disease development time had little impact on the significant metabolites. In regard to the prediction power of the signature, we found it to be more robust than the 77% achieved with previous predictive decision tree modeling conducted with a smaller subset of the SWIFT cohort [19]. The predictive power of the metabolic signature was also superior to well-known clinical diagnostics, including FPG, 2-hour post-load glucose from the 75-g OGTT (accuracy approximately 71%), HOMA-IR, family history of diabetes, and type of prenatal GDM treatment. We also achieved higher discriminating power than other published metabolomics-based diagnostic studies [19,27,28,41,45,46]. Of potential significance, the metabolite predictive signatures reported here do not rely on accompanying clinical parameters [27,41]. Although clinical parameters such as FPG, 2hPG, and HOMA-IR at baseline were significantly different between the incident T2D case and non-T2D control groups in the present study, they did not yield an improvement in the prediction power when added to our metabolite-based model.

Metabolic profiling here revealed several metabolic pathways and specific metabolites associated with the development of and progression to T2D among women with previous GDM pregnancy. Hexose, which represents the sum of all 6-carbon monosaccharides including glucose and fructose, was the only metabolite with a significant increase in all 3 analyses (baseline, follow-up, and longitudinal). The association between hexose and future T2D risk was highly significant (FDR < 0.001) at baseline, consistent with several other prospective cohort studies [19,41,47–49]. Hexose was also significantly increased, as expected, in women who developed T2D within 2 years of follow-up [24,50]. More importantly, while women who subsequently developed T2D already had higher hexose levels at baseline, their levels further increased over the observation period compared to those who remained normoglycemic (Fig 5B). However, it is not surprising that hexoses (likely glucose) are elevated, since diabetes and its severity are defined by the level of circulating glucose. Although there is constant interplay between nutrient metabolism pathways, carbohydrate metabolism takes precedence over protein and fat in terms of energy generation under most circumstances. The perturbations in circulating hexoses may indicate problems with carbohydrate metabolism but may also point to an underlying problem with AA or lipid metabolism.

BCAAs (isoleucine, leucine, and valine) and aromatic AAs are reported to positively associate with insulin resistance, future development of T2D (incident), and existing T2D (prevalent) [24,25,41,51–55]. In line with these studies, we found that the elevation of BCAAs and tyrosine together was associated with subsequent onset of overt diabetes at baseline and existing T2D at follow-up (Fig 6A). The fact that BCAAs are elevated is likely suggestive of increased absorption or production in the gut microbiome, or reduced utilization/breakdown/degradation. In addition to elevated BCAAs, and more importantly, there was a positive association between a broad spectrum of AAs and T2D risk, possibly suggesting that AA catabolism was attenuated. Glucagon regulates AA catabolism, and disruption of glucagon receptor (GCGR) signaling is associated with an increase in circulating AAs [56–58]. Therefore, our finding of an overall increase in circulating AAs is consistent with aberrant GCGR signaling, which has been reported in T2D patients previously [59].

The complex relationships among phospholipids and sphingomyelin metabolites and diabetes risk have not been extensively studied. In the present study, at both baseline and follow-up, 6 diacyl-glycerophospholipids (the PC aa C group) were positively associated with T2D, while 11 acyl-alkyl-glycerophospholipids (the PC ae C group) were inversely related with T2D risk (Fig 6A). Several sphingomyelins were also shown to be negatively associated with diabetes risk (Fig 6A). In a case–cohort study in the framework of the European Prospective Investigation into Cancer and Nutrition (EPIC)–Potsdam, replicated in KORA, some diacyl-phosphatidylcholines were indeed shown to be associated with higher risk of T2D, while sphingomyelins and 1 acyl-alkyl-phosphatidylcholine (C18:2) were associated with lower risk [41]. More importantly, in a subset of the Hyperglycemia and Pregnancy Adverse Outcome (HAPO) study, disturbed lipid metabolism in particular classes of phospholipids and lysophospholipids were detected prior to hyperglycemia at 2 years postpartum in diabetes risk groups (GDM in previous pregnancy and glucose value higher than normal but below GDM criteria) [60]. Our study, with a significantly larger number of analytes measured in a large population, strongly supports the notion that an inverse association between PC aa and PC ae groups and T2D exists. It also suggests a pathway preference for glycerophospholipid metabolism versus ether lipid metabolism in progression to T2D [41,61]. Interestingly, in contrast to the finding of an inverse association between sphingomyelin metabolism and T2D incidence (Fig 6A) in our study and others [19,28,41], some studies showed sphingomyelins were up-regulated in T2D patients, or positively associated with future T2D [62,63]. Thus, the role of sphingomyelins in T2D incidence among different populations needs to be further explored.

In the present study, we also performed longitudinal profiling of 337 individuals, tracking changes in metabolic profiles over the course of diabetes progression. This analysis provides insight into molecular changes that occur during transition from a normoglycemic state to a T2D state. Using a longitudinal panel analysis, 10 metabolites were identified to be significantly dysregulated during T2D progression (Fig 5A). Kynurenine and the kynurenine-tryptophan metabolism pathway, as implicated here, as well as valine, have been suggested to play an important role in obesity, insulin resistance, and diabetes [64–70]. The downward patterns of histidine, serine, citrulline, and glycine observed suggest AA disposal and proteinogenic processes are attenuated in diabetes progression. This could result in the overall increased circulating AA levels. Interestingly, except for these 10 significantly changed metabolites, most were unchanged in the longitudinal analysis (Fig 6A), in contrast with cross-sectional studies of baseline and follow-up time points. The patterns observed in these metabolites, seen as early as 9 weeks postpartum, may indicate the presence of a subclinical dysmetabolism preceding diabetes at baseline, which is supported by our previous epidemiological studies [3,71].

The present study has 3 main strengths. First, a well-characterized prospective cohort without T2D at 6–9 weeks postpartum (study baseline) was studied. Among these women, over 90% completed annual in-person study visits including research 2-hour OGTTs up to 2 years post-baseline and/or had additional testing of glycemia for clinical diagnosis of new onset T2D in KPNC electronic health records within 8 years post-baseline. Fasting plasma samples were collected at SWIFT baseline and at each in-person follow-up study visit up to 2 years post-baseline. Second, the cohort was racially and ethnically diverse, therefore minimizing bias caused by confounders of race/ethnicity. Third, we performed cross-sectional analyses at both baseline and follow-up. More importantly, we performed longitudinal analysis, tracing metabolic changes within individuals, and profiling diabetes progression in each case.

One potential limitation of our study is that all the analyses were performed within the same cohort, although we have samples from the same participants at different time points (baseline and follow-up). In the predictive model build-up, we partitioned the entire group of participants into training (70%) and testing (30%) groups. By using the training set for building the predictive model, which was afterwards validated in the testing set, we minimized the possibility of overfitting. It would be ideal to have an independent cohort in which to validate our biomarker signature and other findings, but this is beyond the scope of this current study. Another limitation is that we could not exclude all potential confounding effects since some were not recorded in the database.

In closing, we observed that women with recent GDM who subsequently progress to T2D indeed have a subclinical dysmetabolism preceding diabetes at 2 months postpartum, when most have returned to normoglycemia. During the transition from GDM to new onset T2D, while glucose levels progressively increase, the underlying mechanism may include defects in the metabolism of AAs and phospholipids, further worsening insulin resistance and hyperglycemia. Those changes in AA and lipid metabolism from the early postpartum period are likely not the consequence of T2D development but may be causally involved in disease onset and progression.

## Supporting information

S1 Fig Generation of predictive models. (A) Workflow of building predictive model. (B) Performance of predictive models (metabolic signature, fasting plasma glucose [FPG], 2-hour plasma glucose [2hPG]) indicated by mean, median, and standard deviation (SD).
(TIF)

S2 Fig Metabolites associated with future type 2 diabetes (T2D). (A) Partial least squares discriminant analysis (PLS-DA) of metabolites at baseline. (B) The cross-validation analysis of PLS-DA. (C) The permutation test of PLS-DA. (D) Abundance of altered metabolites at baseline in incident T2D and non-T2D groups. Rows are metabolites grouped based on hierarchical clustering, and columns are individuals (incident T2D cases are in red on the left and controls are in green on the right). Values were log transformed and scaled.
(TIF)

S3 Fig Metabolites associated with type 2 diabetes (T2D). (A) Partial least squares discriminant analysis (PLS-DA) of metabolites at follow-up. (B) The cross-validation analysis of PLS-DA. (C) The permutation test of PLS-DA. (D) Abundance of altered metabolites at follow-up in T2D and non-T2D control groups. Rows are metabolites grouped based on hierarchical clustering, and columns are individuals (T2D cases are in red on the right and controls are in blue on the left). Values were log transformed and scaled.
(TIF)

S4 Fig Longitudinal study showing 10 metabolite changes during T2D progression. Dot plots showing relative abundance of 10 differential metabolites in individuals in longitudinal analysis at baseline and follow-up. Red indicates progressors and blue indicates non-progressors. The lines represent a mean trajectory of designated metabolites over time.
(TIF)

S1 Table Metabolites significantly altered in incident T2D at baseline. (XLSX)

S2 Table Significance of correlation between 37 differential metabolites and clinical parameters. (XLSX)

S3 Table Pathways significantly regulated in incident T2D at baseline. (XLSX)

S4 Table Metabolites significantly altered in T2D at follow-up. (XLSX)

S5 Table Pathways significantly altered in T2D at follow-up. (XLSX)

S6 Table Significant metabolites in Cox regression model and logistic regression model. (XLSX)

S1 Text Study design and protocol of SWIFT cohort. (DOCX)

S2 Text STROBE checklist. (DOCX)

## Abbreviations

2hPG: 2-hour plasma glucose
AA: amino acid
ADA: American Diabetes Association
AUC: area under the receiver operating characteristic curve
BCAA: branched-chain amino acid
FDR: false discovery rate
FPG: fasting plasma glucose
GDM: gestational diabetes mellitus
HOMA-B: homeostatic model assessment for beta cell function
HOMA-IR: homeostatic model assessment for insulin resistance
KEGG: Kyoto Encyclopedia of Genes and Genomes
KPNC: Kaiser Permanente Northern California
LOD: limit of detection
lysoPC: lysophosphatidylcholine
OGTT: oral glucose tolerance test
SWIFT: Study of Women, Infant Feeding and Type 2 Diabetes after GDM Pregnancy
T2D: type 2 diabetes
