# Supplementary figures and images for: Amino acid and lipid metabolism in post-gestational diabetes and progression to type 2 diabetes: A metabolic profiling study
Source: PLoS Med. 2020 May 20;17(5):e1003112. doi: 10.1371/journal.pmed.1003112 (PMC7239388; doi:10.1371/journal.pmed.1003112)

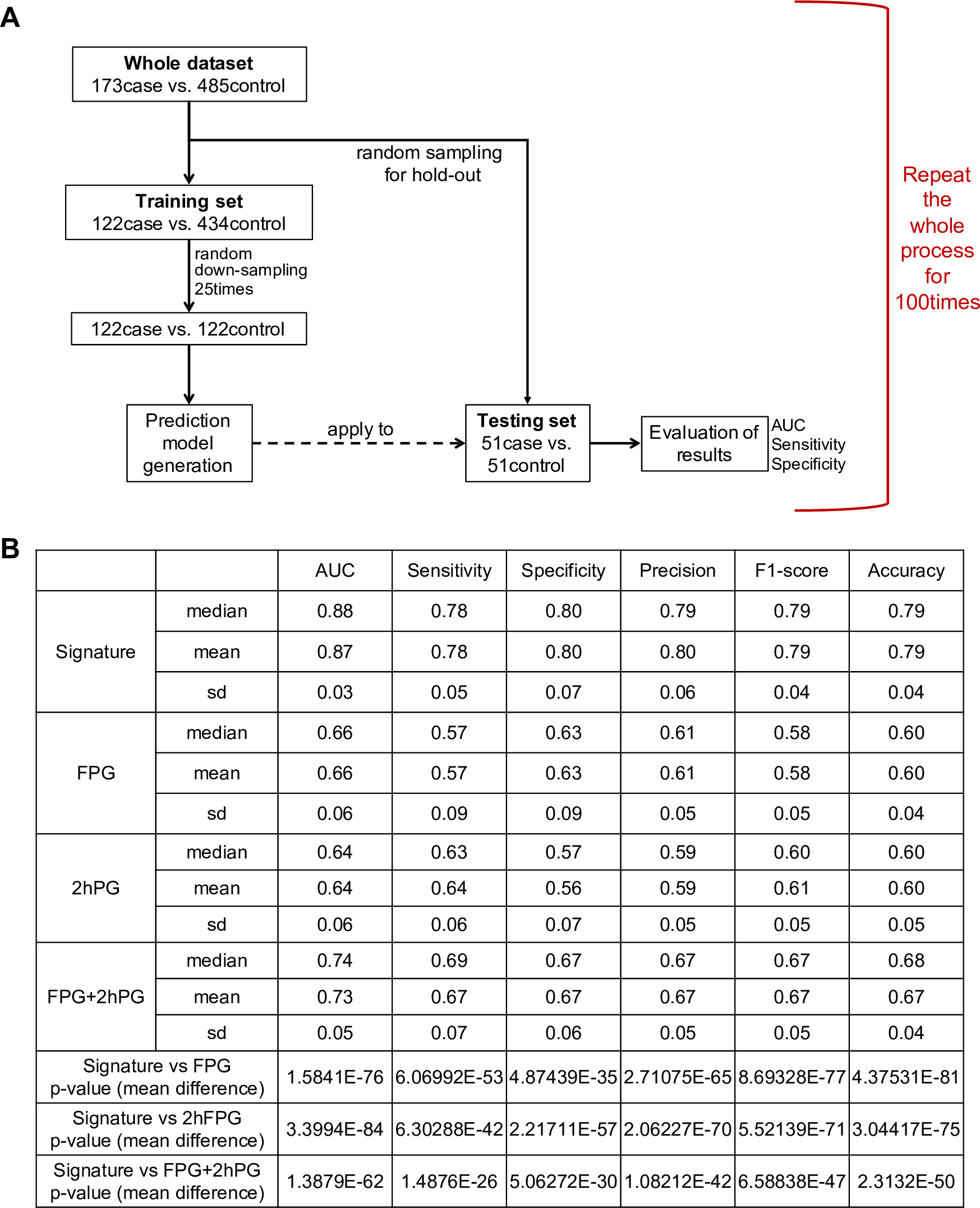

Supplement: S1 Fig — (A) Workflow of building predictive model. (B) Performance of predictive models (metabolic signature, fasting plasma glucose [FPG], 2-hour plasma glucose [2hPG]) indicated by mean, median, and standard deviation (SD). (TIF) [file pmed.1003112.s001.tif]

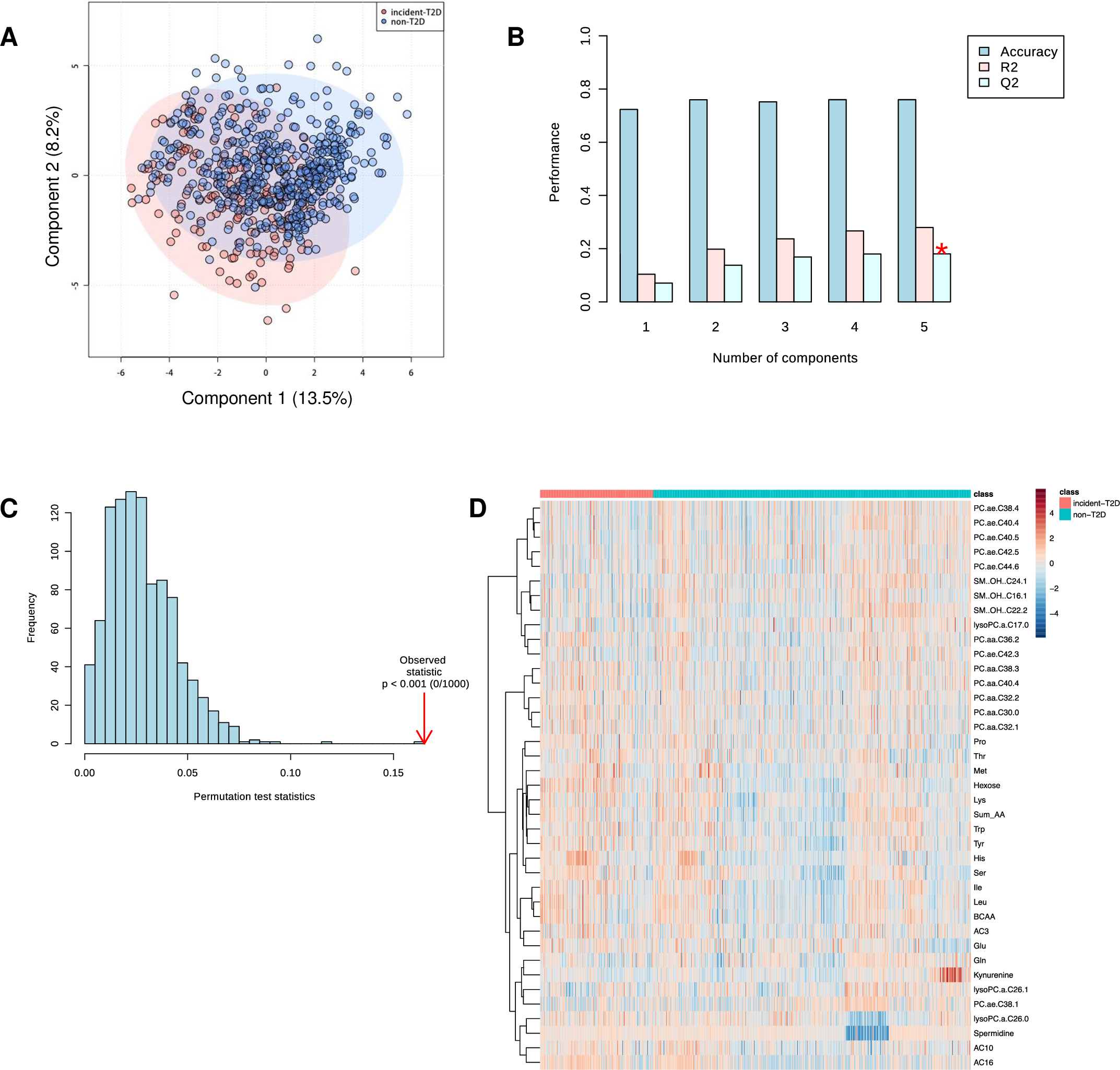

Supplement: S2 Fig — (A) Partial least squares discriminant analysis (PLS-DA) of metabolites at baseline. (B) The cross-validation analysis of PLS-DA. (C) The permutation test of PLS-DA. (D) Abundance of altered metabolites at baseline in incident T2D and non-T2D groups. Rows are metabolites grouped based on hierarchical clustering, and columns are individuals (incident T2D cases are in red on the left and controls are in green on the right). Values were log transformed and scaled. (TIF) [file pmed.1003112.s002.tif]

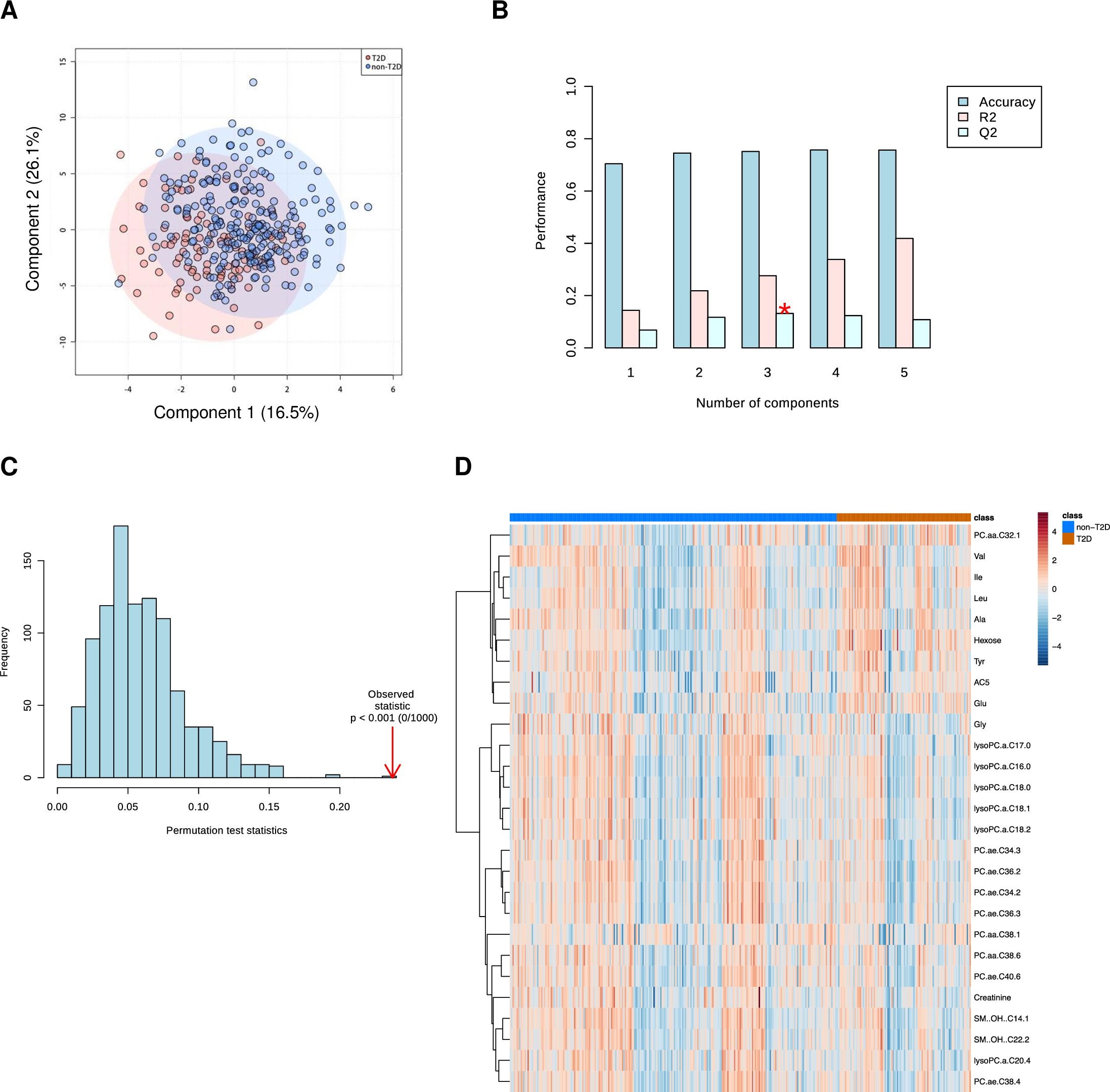

Supplement: S3 Fig — (A) Partial least squares discriminant analysis (PLS-DA) of metabolites at follow-up. (B) The cross-validation analysis of PLS-DA. (C) The permutation test of PLS-DA. (D) Abundance of altered metabolites at follow-up in T2D and non-T2D control groups. Rows are metabolites grouped based on hierarchical clustering, and columns are individuals (T2D cases are in red on the right and controls are in blue on the left). Values were log transformed and scaled. (TIF) [file pmed.1003112.s003.tif]

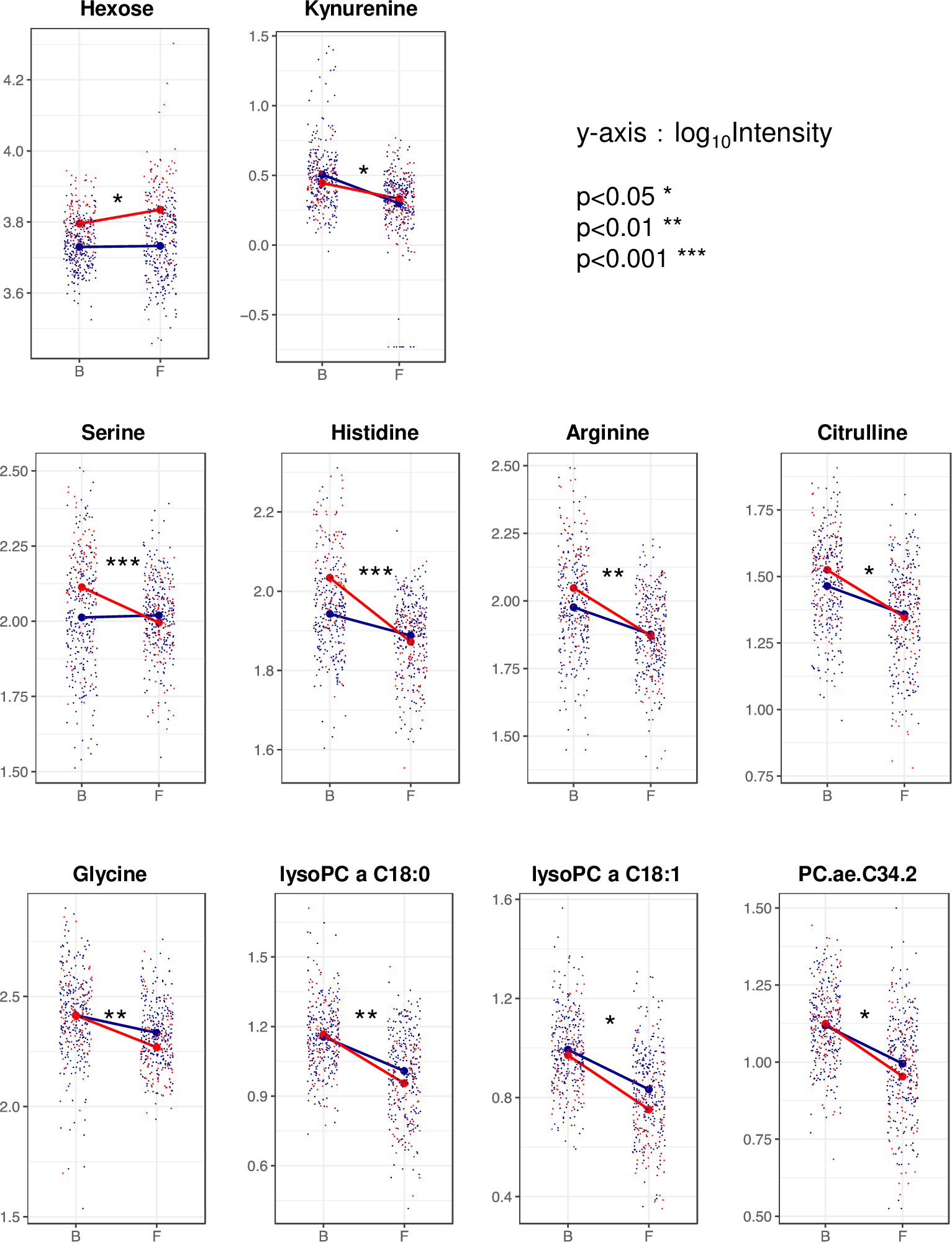

Supplement: S4 Fig — Dot plots showing relative abundance of 10 differential metabolites in individuals in longitudinal analysis at baseline and follow-up. Red indicates progressors and blue indicates non-progressors. The lines represent a mean trajectory of designated metabolites over time. (TIF) [file pmed.1003112.s004.tif]
