# Supplementary material for: Amino acid and lipid metabolism in post-gestational diabetes and progression to type 2 diabetes: A metabolic profiling study
Source: PLoS Med. 2020 May 20;17(5):e1003112. doi: 10.1371/journal.pmed.1003112 (PMC7239388; doi:10.1371/journal.pmed.1003112)
Supplement: S1 Text — (DOCX) [file pmed.1003112.s011.docx]

**SWIFT: Study of Women, Infant Feeding and Type 2 Diabetes After GDM Pregnancy (SWIFT)**

**Study Description**

The goal of the Study of Women, Infant Feeding and Type 2 Diabetes after GDM pregnancy (SWIFT) is to determine the relation of longer and more intensive lactation, as compared to formula feeding, on progression to incident type 2 diabetes mellitus among women within several years following delivery of a GDM pregnancy. The study enrolled women with recent GDM at 6 to 9 weeks post-delivery to classify oral glucose tolerance and evaluate the relation of lactation intensity and duration to persistent changes in blood glucose levels, insulin resistance, and changes in body weight, waist circumference, and adiposity during several years post-baseline. SWIFT is a prospective, observational cohort study of 1,035 women diagnosed with gestational diabetes mellitus (GDM) via Carpenter and Coustan criteria during pregnancy who are at high risk for conversion to prediabetes and type 2 diabetes within 5-10 years post-delivery.

**Study Design**

Study Type: Observational

Actual Enrollment: 1035 participants

Observational Model: Cohort

Time Perspective: Prospective

Official Title: SWIFT: Study of Women, Infant Feeding and Type 2 Diabetes After GDM Pregnancy

Study Start Date: May 2008

Estimated Primary Completion Date: September 2025

Estimated Study Completion Date: September 2025

**Groups and Cohorts**

Women with recent GDM pregnancy

The study cohort includes women who had gestational diabetes mellitus (GDM) in their index pregnancy for study enrollment. There are two pre-defined groups: 1) women who breastfeed intensively during the first 4 months postpartum, and 2) women who mostly fed formula during the first 4 months postpartum. The study enrolled women into these pre-defined groups, but some women transitioned into mixed feeding groups after enrollment.

**Outcome Measures**

Primary Outcome Measures:

1. Incident Type 2 Diabetes [ Time Frame: baseline to 10 years postpartum]

Two-hour 75gram oral glucose tolerance test; fasting plasma and 2-hour post-load plasma samples analyzed for glucose and insulin concentrations.

Secondary Outcome Measures:

1. Maternal weight [ Time Frame: 2 years postpartum]

Body weight

1. Body composition [ Time Frame: 2 years postpartum]

Tetra polar Bioelectrical impedance to estimate percent body fat

1. Maternal waist circumference [ Time Frame: 2 years postpartum]

Waist circumference

Other Outcome Measures:

1. Insulin Resistance Index [ Time Frame: 2 years postpartum]

Fasting plasma and 2hour post-load plasma assayed for concentrations of glucose and insulin. These measures will be used to calculate homeostatic model assessment of insulin resistance (HOMA-IR).

1. Insulin secretion Index [ Time Frame: 2 years postpartum]

Fasting plasma and 2hour post-load plasma assayed for concentrations of glucose and insulin. These measures will be used to calculate the homeostatic model assessment of insulin secretion (HOMA-ß).

Biospecimen Retention:   Samples With DNA

We collected fasting and 2-hour plasma specimens during each Oral Glucose Tolerance Test (OGTT). The EDTA treated plasma specimens and buffy coats are stored in cryovials at -70 degrees Centrigrade in low temperature freezers.

**Eligibility Criteria**

Ages Eligible for Study: 20 Years to 45 Years (Adult)

Sexes Eligible for Study: Female

Accepts Healthy Volunteers: No

Sampling Method: Non-Probability Sample

**Study Population**

SWIFT participants were recruited from 13 Kaiser Permanente Northern California (KPNC) medical facilities throughout the region. Participating field sites for data collection were located within the three primary areas including:

North area: Sacramento, South Sacramento, and Roseville Medical Centers, and Rancho Cordova, Elk Grove, Point West, and Folsom Medical Offices; East area: Division of Research (DOR) Research Clinic (Oakland), Hayward Medical Center and Fremont Medical Offices and Richmond Medical Center; South area: Santa Clara, and San Jose Medical Centers. The prospective cohort enrolled women who received prenatal care and delivered a singleton, live born infant of 35 weeks gestation or longer at a Kaiser Permanente hospital between July 2008 and October 2011.

**Criteria**

Inclusion Criteria:

- age 20 to 45 years at delivery
- received prenatal care in Kaiser Permanente Northern California (KPNC) health care system
- GDM pregnancy diagnosed using the 3-hour 100 g OGTT by Carpenter and Coustan criteria
- delivered a singleton, live birth >= 35 weeks gestation
- no pre-existing diabetes or other serious medical conditions prior to index GDM pregnancy
- no diabetes diagnosis (2-hour 75 gram OGTT) at 6 to 9 weeks postpartum for the index GDM pregnancy
- no use of thyroid medications, steroids, or other medications affecting glucose tolerance
- not planning to move from the northern California area within the subsequent 24 months
- not planning another pregnancy within the next two years
- Two infant feeding groups: women who did not lactate or did so for less than 3 weeks, OR women who provided no supplemental milk feeds at 2-4 weeks and planned to continue intensive lactation defined as <= 1 formula supplement (6 oz/day) from 6-9 weeks until 4 months or more postpartum.

Exclusion criteria:

- women who fed breast milk and 7-16 oz of formula (mixed feeding) during the first 4 weeks of life

**Contacts and Locations**

**Locations**

**United States, California**

Kaiser Permanente Northern California, Division of Research

Oakland, California, United States, 94612

**Sponsors and Collaborators**

Kaiser Permanente

Eunice Kennedy Shriver National Institute of Child Health and Human Development (NICHD)

National Institute of Diabetes and Digestive and Kidney Diseases (NIDDK)

American Diabetes Association

W.K. Kellogg Foundation

Centers for Disease Control and Prevention

National Heart, Lung, and Blood Institute (NHLBI)

**Investigators**

Principal Investigator: Erica P Gunderson, PhD Kaiser Permanente
